# Depleting NFAT1 expression inhibits the ability of invasion and migration of human lung cancer cells

**DOI:** 10.1186/1475-2867-13-41

**Published:** 2013-05-12

**Authors:** Ji-fu Liu, Shou-hua Zhao, Shan-shan Wu

**Affiliations:** 1 Department of thoracic surgery, General Hospital of Beijing Military Region, Nan Men Cang 5, Dongcheng District, Beijing, China

**Keywords:** Lung cancer, NFAT1, Pathogenesis, RNA interference, Invasion assay

## Abstract

**Background:**

Nuclear factor of activated T-cells (NFAT) is a general name applied to a family of transcription factors shown to be important in immune response. One or more members of the NFAT family are expressed in most cells of the immune system. NFAT1 is considered to involve in the development of cardiac, skeletal muscle, nervous systems, and tumorigenesis.

**Methods:**

In the current study, we analyzed MEKK1 expression in 159 surgically resection non-small cell lung cancer patient’s samples by immunohistochemistry and determined its role in SK-EMS-1 cells via RNAi experiment.

**Results:**

The abilities of invasion, motility, and adhesion of SK-EMS-1 cells were detected by transwell assay, wound healing assay and adhesion assay, respectively. The result showed NFAT1 was highly expressed in lung tumor tissues instead of adjacent lung tissues (54.1% vs 8.8%, p < 0.05); its overexpression was positively correlated with lymph node metastasis (p < 0.05). Depleting its expression in SK-EMS-1 cells can inhibit its invasion and migration abilities significantly (p < 0.05); and also can reduce proliferation of lung cancer cells (p < 0.05).

**Conclusion:**

Our study showed NFAT1 plays an important role in origination, invasion and metastasis of non-small lung cancer cells; its underlying action mechanism needs further study.

## Introduction

Nuclear factor of activated T cells (NFAT) was initially identified as an inducible nuclear factor that could bind the interleukin-2 (IL-2) promoter in activated T cells [1]. When all of the proteins of the NFAT family had been isolated and molecularly characterized, it became clear that their expression was not limited to T cells. At least one NFAT family member is expressed by almost every cell type that has been examined, including other cells of the immune system and non-immune cells [2-5]. The NFAT family consists of five members: NFAT1 (also known as NFATp or NFATc2), NFAT2 (also known as NFATc or NFATc1), NFAT3 (also known as NFATc4), NFAT4 (also known as NFATx or NFATc3) and NFAT5, all are calcium responsive and are regulated by the calcium/calcineurin signaling pathway [3,6]. The terms NFATp and NFATc were originally used to describe DNA-binding activities present in cellular extracts; hence, more than one NFAT family protein may have been present in the original NFATp (preexisting) and NFATc (cytoplasmic) preparations [7].

In resting cells, NFATs are phosphorylated at a cluster of serine residues located in the regulatory domain, effectively masking a nuclear localization signal, thereby retaining NFAT in an inactive conformation in the cytoplasm [2,8], Upon stimulation with agonists that elicit an increase in intracellular calcium, NFATs are dephosphorylated by the phosphatase calcineurin and translocate to the nucleus. Here they are transcriptionally active by binding to the promoter regions of target genes [9,10]. Classical NFATs typically interact with other transcription factors such as AP-1 [11,12] and GATA4 [13] to activate transcription. Adjacent NFAT and AP-1 binding sites are present in the promoter region of inducible genes including IL-2 and cyclooxygenase-2 (COX-2) [11,14]. Recent studies have showed the expression of NFAT in human cancers, such as breast Cancer [15] and human metastatic melanoma cell lines [16], however, the expression of NFAT in non-small-cell lung cancer (NSCLC) remain undefined. Therefore, the aim of this study was to investigate its role in origination, invasion and metastasis of lung cancer. We firstly analyzed the NFAT1’s expression in lung cancer tissues, and further constructed eukaryotic expression system and RNA interference was introduced to get stable expression lung cancer cell lines with or without NFAT1 to discuss its effect on lung cancer cell growth and invasion.

## Materials and methods

### Patients and samples

The paraffin-embedded tissue samples from 159 patients with non-small cell carcinoma diagnosed between 2008 and 2011 were obtained from the department of Thoracic Surgery, General Hospital of Beijing Military Region. All the tissue samples were obtained after resectional operation of the lung cancer. Information of age, gender, histological type, differentiation grade, and lymph node metastasis of lung adenocarcinomas were obtained from the Surgical Pathology Files in the hospitals. The clinicopathological diagnosis on the tumor status was evaluated by the clinical pathologists in the hospital.

### Immunohistochemistry

Standard indirect immunoperoxidase procedures were used for immunohistochemistry. In brief, the sections of 159 patients were incubated in NFAT1 primary monoclonal antibody (43-γ, Santa Cruz) (1:200) overnight at 4°C. Control sections were incubated with PBS instead. After washing four times with PBS, the sections were incubated in biotinylated anti-rabbit IgG (1:1,000) for 1 h, followed by rinsing and incubation with avidin–biotin–peroxidase complex for 30 min. The peroxidase reaction was developed using 3, 3-diaminobenzidine for 6 min. The immunostained slides were evaluated independently by two pathologists. The brown staining in cytoplasm was defined as the positive signal.

### Cell Lines and specimens

The human lung fibroblasts (HLF-1), human non-small cell lung cancer cell lines A549 and SK-MES-1 were obtained from ATCC(Massachusetts, USA), they were cultured in RPMI-1640 media supplemented with 10% fetal calf serum, penicillin (100 U/mL) and streptomycin (100 ug/mL) (Gibco BRL), and incubated at 37°C in a humidified air atmosphere containing 5% CO2. The cells were harvested with three time washing.

### Real Time PCR

Total RNA was extracted with Trizol (Invitrogen). RT-PCR was performed with 1 μg of total RNA after the first chain of cDNA synthesized with a condition of an initial denaturation at 95°C, denature at 94°C for 30 s, annealing at 59°C for 45 s and elongation at 72°C for 1 min for 30 cycles. The primers are, NFAT-1 (137 bp): forward: TTCCTACCCCACAGTCATTC and reversed: AGGTCTGGTCCAAGTTCTG; GAPDH (590 bp): forward: ACCCAGAAGACTGTGGATGG and reversed: AGGGGTCTACATGGCAACTG. The primers for both NFAT1 and GAPDH were used in the same reaction during the PCR amplification. qRT-PCR carried out using SYBR Green master mix with 7900HT Sequence Detection System from Applied Biosystems (USA). The mRNA expressions of NFAT-1 were compared among HLF-1, A549 and SK-MES-1.

### NFAT1-siRNA and cell transfection

Small interfering RNAs (siRNAs) targeting NFAT1 (U43342 in GenBank) were chemically synthesized (GenePharma Co.). The target sequences for effective silencing of NFAT1 gene were selected from 1,620 to 1,638nt (siRNA1, sense sequence: 5′CCUUCAGACCCAAUGUUAUTT 3′; antisense: 5′AUAACAUUGGGUCUGAAGGTT3′), 1,686–1,704nt (siRNA2, sense sequence: 5′GCUGCAGCGUUCUGUCAAUTT 3′, antisense: 5′ AUUGACAGAACGCUGCAGCTT 3′); and 2,898–2,916nt (siRNA3, sense sequence: 5′ CCGGGUGUUUCAACUAGAATT 3′; antisense: 5′ UU CUAGUUGAAACACCCGGAG 3′). The over-hanged UU in the siRNAs was replaced by dTdT for enhancement of its stability in cells. A total amount of 1.6-μg siRNA per well was used to transfect the SK-MES-1 cells using LipofectAMINE 2000 (Invitrogen) according to the manufacturer’s protocol. A non-specific gene sequence was used as a negative control. The GFP-conjugated control siRNA was used to evaluate the transfection efficiency. RT-PCR and Western blot were used to analyze NFAT1 mRNA and protein level, respectively.

### Western blotting

Total cellular protein was extracted using lysis buffer [50 mM Tris–HCl (pH 7.4), 1 mM EDTA, 250 mM NaCl, 1% Triton X-100, 5 mM MgCl_2_, 2 mM Na_3_VO_4_, and 1× Complete™ protease inhibitor]. Protein concentration was measured using Bio-Rad protein assay. Equal amount of protein was separated using 10% SDS-PAGE and transferred to nitrocellulose membranes (Amersham). The membranes were blocked with 5% skimmed milk in PBS buffer and incubated overnight with primary antibodies followed by horseradish peroxidase conjugated antibodies at room temperature. β-actin was used as internal positive control. Primary antibodies included NFAT1 (C-22) was purchased from Santa Cruz. Signals were visualized using enhanced chemiluminescence system (Amersham).

### Invasion assay

Invasion assay was performed using Boyden chamber system (Neuro Probe) with a fibronectin-precoated (0.5 mg/ml) polycarbonate membrane (8 μm pore size). The lighter side of the polycarbonate membrane was precoated with 250 μg/ml matrigel (BD). The bottom chambers were filled with 30-μl RPMI1640 medium containing 2% BSA while the top chambers were filled with 50-μl RPMI1640 serum-free medium containing 0.2% BSA. 5 × 10^4^ cells per well were added to the top chamber, followed by a 15 h incubation in 37°C, 5% CO_2_ incubator. Three independent experiments were performed with triplicate treatment. The cells were fixed in methanol and stained with haematoxylin. The top surface of the membrane was gently scrubbed with a cotton bud, the cells migrated to the lower side of the membrane was counted under the microscope and the numbers of migrated cells were calculated as average plus SD.

### Adhesion assay

The same amounts of NFAT1-siRNA and control-siRNA transfected and untransfected SK-MES-1 cells (3 × 10^4^) were plated onto the matrigel-precoated (50 μg/ml) 96-well plate in triplicate. The cells were washed at 30, 60, and 120 min to remove non-adherent cells. 0.2% BSA precoated wells served as the negative control. After washing, the adhered cells were measured with MTT assay. The relative optical density (OD) was determined at 570 nm using WELLSCAN MK3 ELISA (Labsystems, Dragon, Finland) and a 450 nm reference filter. The OD values reflected the proportion of cells in the matrigel coated 96-well plate.

### Wound healing assay

Wound healing assay was conducted as previously described. The distance of wound closure (compared with control at 0 h) was measured in three-independent wound sites per group. Relative cell motility was calculated as the wound width at *t* = 0 h minus the wound width at *t* = 24 h, as indicated. Values from at least three-independent experiments were pooled and expressed as means ± SD.

## Results

### Expression of NFAT1 protein in lung adenocarcinomas and its clinic-pathological significance

**Figure 1:**
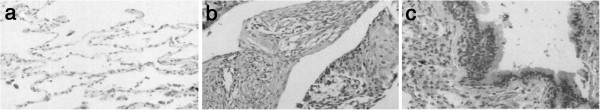
**Immunohistochemistry result of NFAT1 in lung cancers and adjacent tissues (200×). a**, normal tissue (-); **b**, tumor tissue (-); **c**, NFAT1 (+).

Of the 159 non-small cell carcinoma patients, the results of immunohistochemistry demonstrated that the NFAT1 positive staining (Figure 1c) was noted in 86 samples (54.1%) while only 14 positive cases in adjacent normal lung tissues (8.8%), p < 0.01. These results suggested NFAT1 is positively correlated with origination of lung cancer. We further analyzed the relationship of the NFAT1 positive staining with the progression and invasion of the tumor. We observed that all the samples with the lymph node metastasis were 63.6% positive (N = 55) staining while 13 out of 31 cases of non-lymph node metastasis (42.5%) were of positive staining, p = 0.027 (Table 1). All these results suggested that the NFAT1 expression was significantly associated with lymph node metastasis in the observed non-small lung cancer patients (Table 1; P < 0.05). The NFAT1 staining did not show significant difference among the patients of different gender, age, tumor location, and tumor size (data not shown; *P* > 0.05).

**Table 1:** Expression of NFAT1 protein in 159 non-small cell lung cancer patients

**Parameters**	**Case number**	**P value**
NFAT1 positive in lung cancer tissues	86(159)	
NFAT1 positive in adjacent normal tissues	14(159)	<0.01
Positive in lymph node metastasis	35(55)	0.027
Positive in non-lymph node metastasis	13(31)	

Analyzed by Person’s chi-square test.

### Expression of NFAT1 in lung cancer cell lines

**Figure 2:**
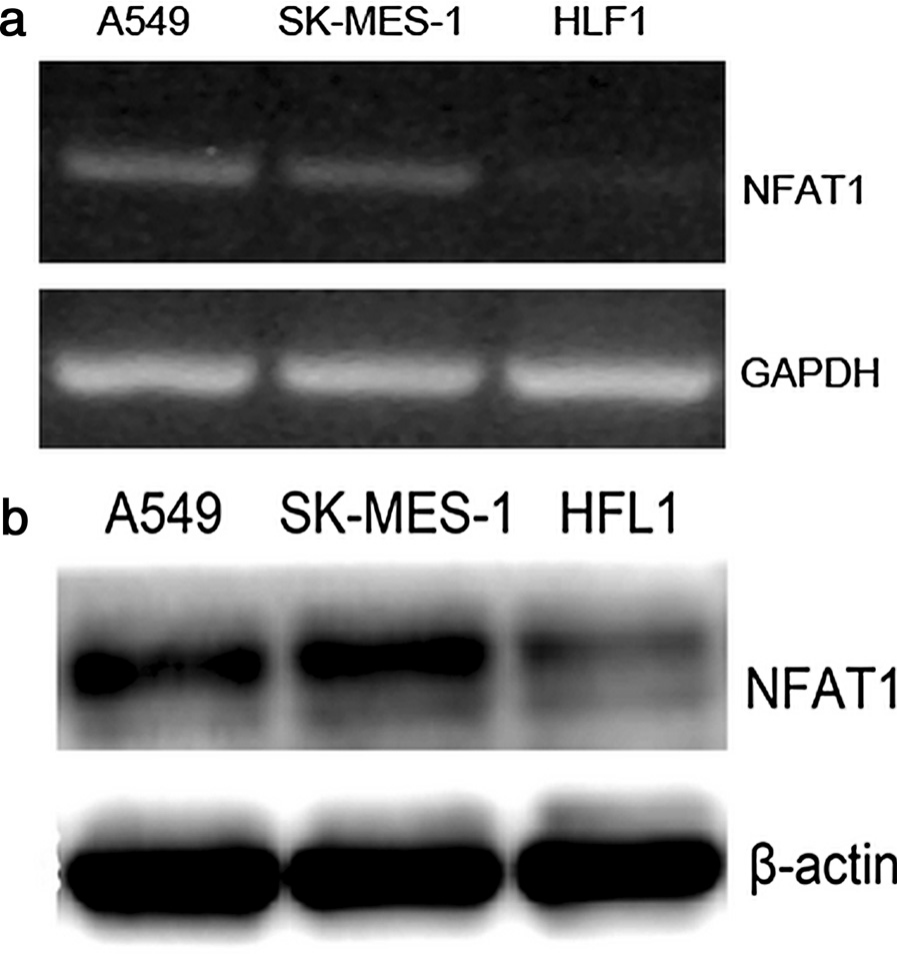
**Expression of NFAT1 in A549, SK-MES-1 and HFL1 cell lines. a**, RT-PCR; **b**, western blotting.

To observe whether the NFAT1 expression is related to lung cancer in vitro, we examined the NFAT1 mRNA and protein levels in different lung cancer cell lines (A549 and SK-MES-1), HLF-1 was used as negative control. The data showed that the NFAT1 mRNA level remained highly abundant in both cell lines compared to HLF-1 (Figure 2a). A Western blot analysis further confirmed the NFAT1 protein expression in a similar pattern in the both observed lung cancer cell lines (Figure 2b).

### Specific depletion of NFAT1 expression by NFAT1 siRNAs

**Figure 3:**
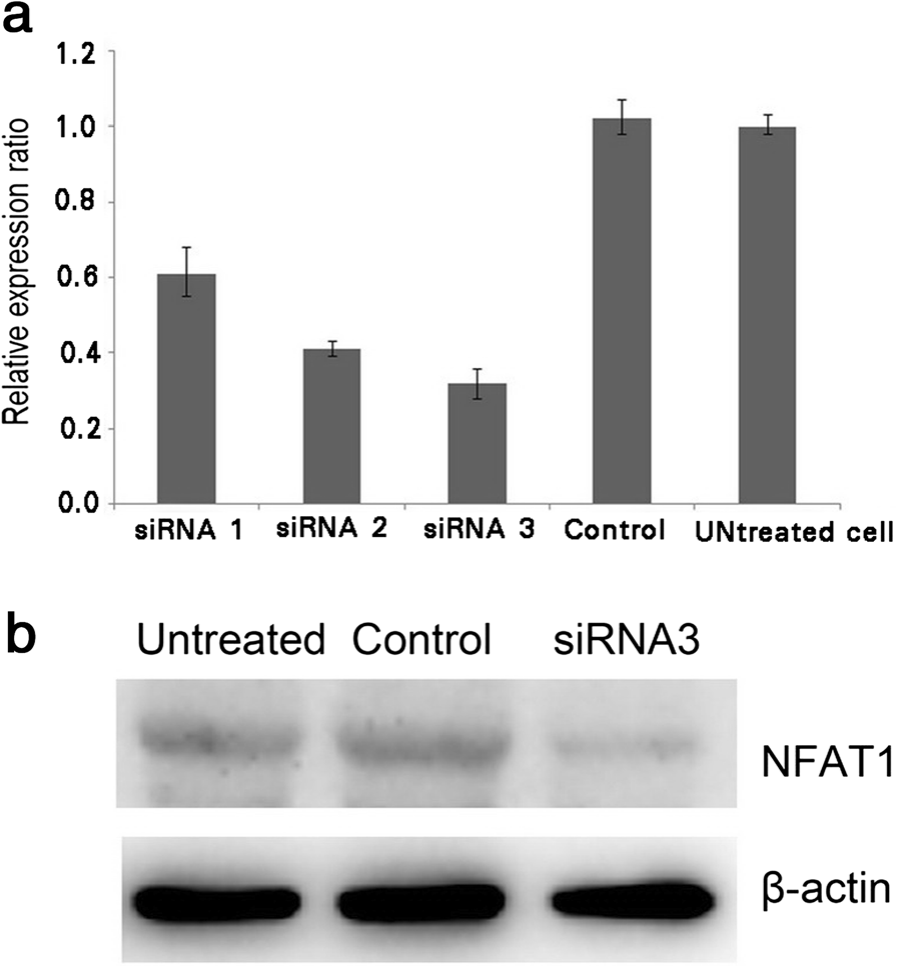
**Expression of NFAT1 in SK-MES-1 cells. a**, RT- PCR result with or without siRNAs; **b**, Western blot with or without siRNA3. GAPDH served as control.

To examine whether the highly expressed NFAT1 is the cause for the strong invasive ability, we depleted NFAT1 using the RNA interference method in SK-MES-1 cells. We selected and synthesized three siRNAs against NFAT1. The efficiency of the selected siRNAs was examined by transfection in the SK-MES-1 cells. RT-PCR analysis revealed that siRNA2 and siRNA3 have a significant inhibitory effect on the endogenous NFAT1 expression while the siRNA1 showed relatively weaker effect (Figure 3a). The effects of the selected siRNA3 were further confirmed by a western blot to prove it could inhibit NFAT1 expression strongly on protein level (Figure 3b). Based on these results, we determined to use siRNA3 for the following experiments.

### Effects of siRNA3 targeting NFAT1 on invasive, motive and adhesive abilities and proliferation of SK-MES-1 cells

**Figure 4:**
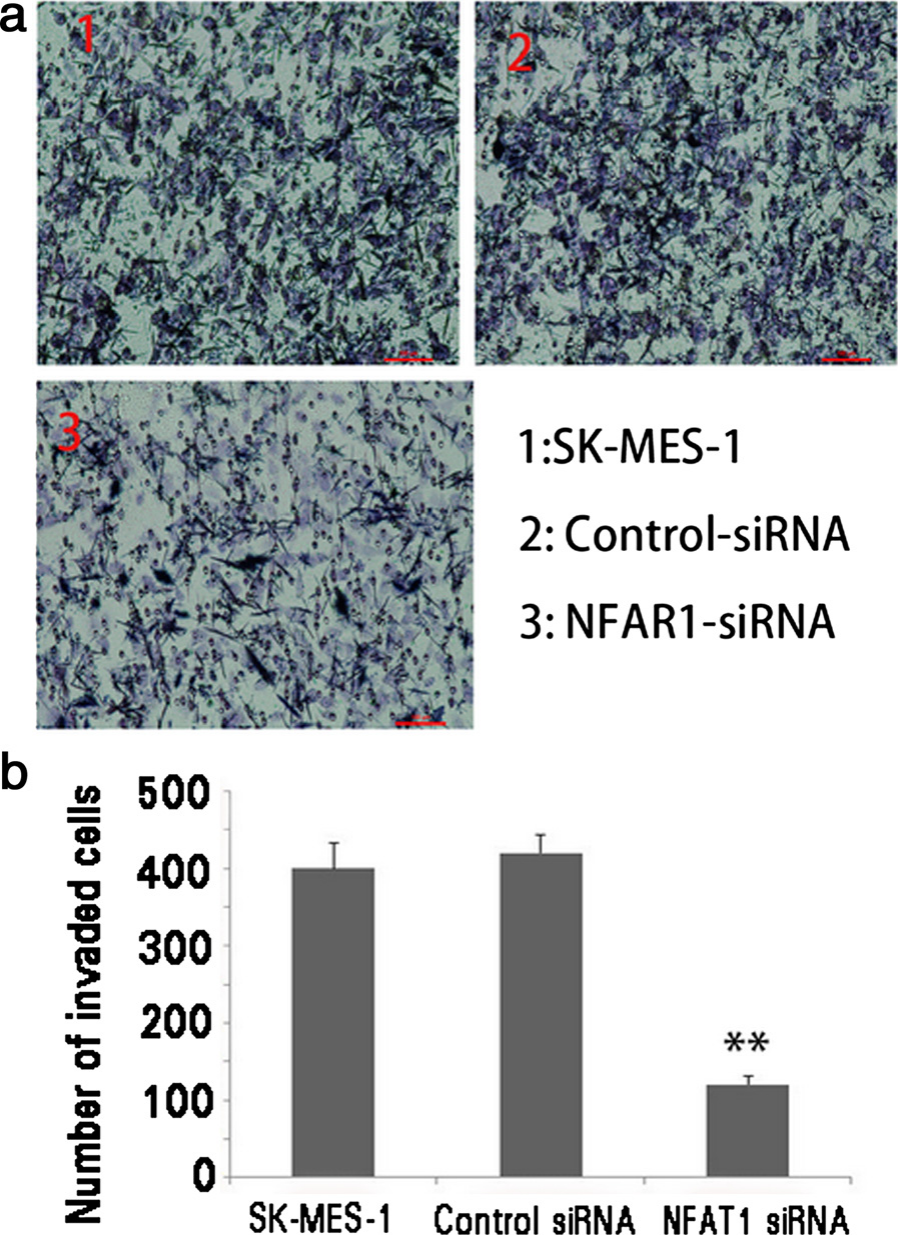
**Depletion of MEEK1 in SK-MES-1 cells. a** Invasion of untransfected cells, control-siRNA, and NFAT1-siRNA3 transfected SK-MES-1 cells; **b** histogram comparison results.

To study the role of NFAT1 on the cell invasion, motility, and adhesion, which are the major characteristics of the metastasis, we used siRNA3, the most powerful one we selected, to deplete the endogenous expression of NFAT1 in the SK-MES-1 cells. All the assays were performed after 48 hour transfection. In an invasion assay, we calculated the number of cells that migrated to the bottom side of the membrane on a chamber where the cells were seeded (Figure 4). The data showed that the wild type SK-MES-1 cells had more numbers of the migrated cells with or without transfection of a control siRNA. However, when siRNA3 targeting NFAT1 was transfected into the cells, numbers of the migrated cells decreased dramatically (Figure 4b). These results suggested that the depletion of NFAT1 significantly suppressed the migration ability of SK-MES-1 cells.

**Figure 5:**
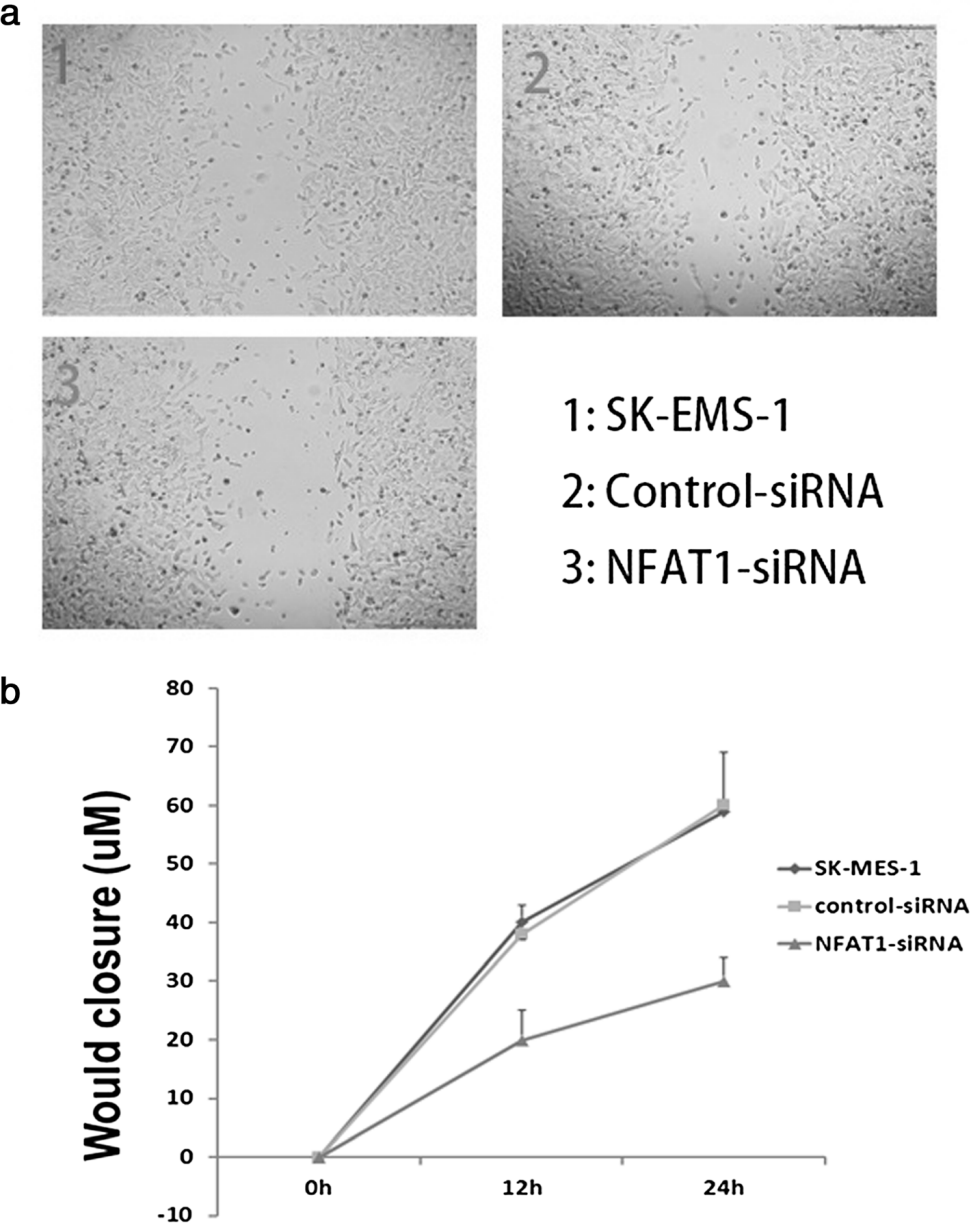
**Depletion of MEEK1 in SK-MES-1 cells. a**, Wound healing result of untransfected cells, control-siRNA and NFAT1-siRNA3 transfected SK-MES-1 cells; **b**, Cell motility results.

To examine whether the depletion of NFAT1 has any effect on the motive ability of the cells, we performed a wound-healing experiment using SK-MES-1 cells transfected with or without control-siRNA and siRNA3. The data showed that the wild type SK-MES-1 cells had no difference in the relative wound closure with or without transfection of the control siRNA, but a significant difference was observed for the cells transfected with or without siRNA3 targeting NFAT1 (Figure 5) both in 15 and 24 h.

**Figure 6:**
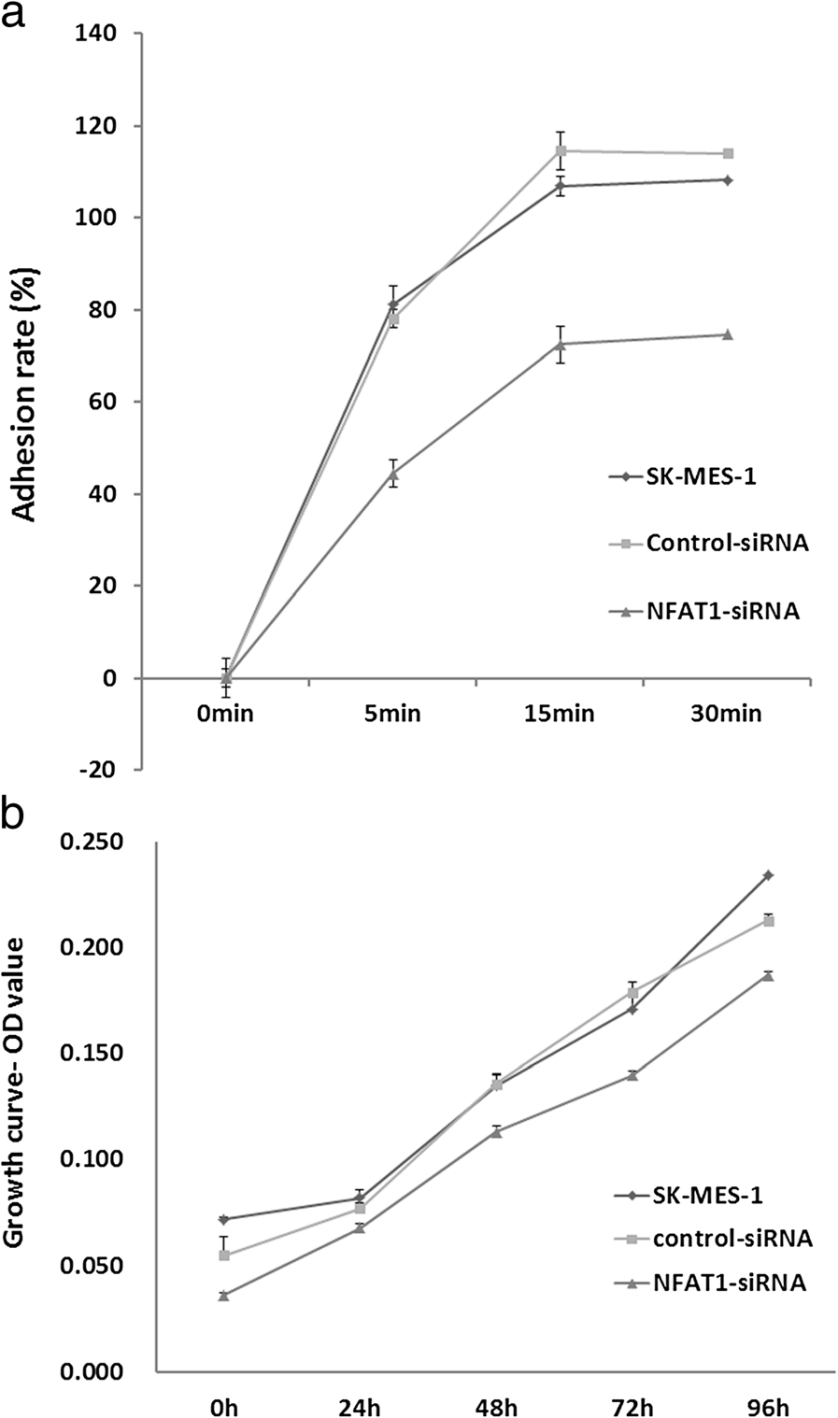
**The adhesion and proliferation test after transfected siRNA3 targeting NFAT1 in SK-MES-1 cells. a**. adhesion ability test; **b**. proliferation ability test. *P < 0.05, **P < 0.01, compared with control.

The highly expressed NFAT1 correlated to the lung cancer metastasis in the tumor patients reminded us to examine whether NFAT1 has an effect on cell adhesion to ECM (extracellular matrix). For this purpose, we performed a cell adhesive assay using siRNA3 targeting NFAT1 in the SK-MES-1 cells. The results showed that the cells, when transfected with siRNA3, obtained low adhesion ability, compared with the wild type cells (Figure 6). The results were in consistence during different times as we observed in 30, 60, and 120 min, respectively. These results indicated that NFAT1 may help the tumor cells adhere to the ECM. Taking together, our data suggest that NFAT1 exerts its effects on metastasis by inhibiting invasion, migration, and adhesion in SK-MES-1 cells.

## Discussion

Failure of treatment for non-small lung cancers is mainly caused by metastasis and invasion of the tumor cells to the neighboring organs. 80% of lung cancer patients were diagnosed at advanced stage. To reveal the metastasis mechanisms is important to help and improve the success of this cancer. Till date, several factors and signaling pathway have been identified in the regulation of metastasis of lung cancer. However, it remains unclear its detailed mechanisms. In this report, we observed that NFAT1 was highly expressed in the lung cancer patients. This is in consistence with the results observed in the lung cancer cell lines. In the current study, we observed that the expression of NFAT1 is closely related to occurrence of lung cancer in the 159 patients by IMC, and more importantly, its overexpression was significantly related with lymph node metastasis.

Gaudineau et al. [17] have conform NFAT1 is a transcription factor that elicits breast carcinoma cells to become invasive, contributing thus to formation of metastasis. To demonstrate whether NFAT1 contributes to metastasis of lung cancer, we depleted it in SK-MES-1 cells, which showed high level of NFAT1 expression and strong invasive ability, and confirmed that NFAT1 plays a major role in controlling the cell invasion, migration, and motility. All of our results are in consistence, suggesting targeting NFAT1 could be a strategy for the development of lung tumor therapy.

NFAT1 initially was determined to transcriptionally regulate cytokines during immune response. NFAT1 is Ca^2+^-responsive and modulated by calcineurin. In the nucleus, they are transcriptionally active and bind to the promoter regions of a set of genes including *TNF-α, IL-2, IL-3, IL-4, IL-5, GM-CSF*, and *IFN-γ*. When cells return to the quiescent state, NFATs are rephosphorylated by kinases, such as protein kinase A and glycogen synthase kinase-3β. As the most studied NFAT, NFAT1 has been shown to elicit both breast cancer and colon carcinoma cell invasion via COX-2. Activation of NFAT1 can also increase vascular smooth muscle cell motility. In the current study, we confirm the importance of NFAT1’s involvement in cell motility; we assume it derives from its participation in PlGF-induced myelomonocytic cell recruitment, and the NFAT1 expression was significantly associated with differentiation, lymph node metastasis in the observed non-small lung cancer patients.

In summary, tumor invasion requires both tumor cell migration and degradation of local matrix barriers. Obviously, expression NFAT1 can enhance these abilities for lung cancer cells. But its detailed action mechanisms need further study.

## Abbreviations

NFAT: Nuclear factor of activated T-cells; NSCLC: Non-small-cell lung cancer.

## Competing interests

The authors have no competing interest to declare.

## Authors' contributions

The work presented here was carried out in collaboration between all authors. J-f L defined the research theme. S-h Z designed methods and experiments; S-s W carried out the laboratory experiments, analyzed the data, interpreted the results and wrote the paper. All authors read and approved the final manuscript.
